# The histopathological results of vestibulectomy specimens in localized provoked vulvodynia in Turkey

**DOI:** 10.11604/pamj.2020.37.267.21240

**Published:** 2020-11-24

**Authors:** Suleyman Eserdag, Didem Kurban, Mine Kiseli, Murat Alan, Yasemin Alan

**Affiliations:** 1 Hera Clinic, Istanbul, Turkey,; 2 Hera Clinic, Ankara, Turkey,; 3 Faculty of Medicine, Ufuk University, Ankara, Turkey,; 4 Tepecik Educational and Research Hospital, Kazim Dirik District, 35100 Bornova, Izmir, Turkey,; 5 Izmir Metropolitan Municipality Esrefpasa Hospital, Izmir, Turkey

**Keywords:** Localized provoked vulvodynia, vestibulitis, vestibulectomy, vulvar intraepithelial lesion, Turkey

## Abstract

**Introduction:**

Localized Provoked Vulvodynia (LPV) is a gynecological disease that is difficult to manage. Despite the wide spectrum of pathophysiological mechanisms and treatment modalities, there is limited success in the management of this disease. Surgical treatment is usually performed as the last resort. We aimed to investigate the histopathological results of 38 women with LPV who underwent surgical vestibulectomy.

**Methods:**

of the 55 women that were diagnosed with LPV and underwent vulvar vestibulectomy, 38 patients with complete histopathological results were included in this retrospective study.

**Results:**

in 14 patients, the pathological reports revealed Low-Grade Squamous Intraepithelial Lesions (LGSIL) (36.8%) whereas for 21 cases (55.2%), the findings were concordant with vestibulitis. The remaining three patients (7.8%) were diagnosed with lichen simplex chronicus.

**Conclusion:**

the presence of LGSIL in the surgical specimens of LPV cases is noteworthy. In this group of patients, surgical excision may contribute to the prevention of progression into high-grade lesions. The relationship between Human Papilloma Virus (HPV) infections and LPV should be further investigated.

## Introduction

Vulvar pain disorders have always been a challenging issue for gynecologists, and idiopathic vulvar pain has been investigated since the 1880s. Vulvodynia is defined as vulvar discomfort that occurs in the absence of relevant visible findings or a specific, clinically identifiable, neurological disorder. The vulvar vestibule is an anatomic region in the entrance of vagina, surrounded by the clitoral frenulum (superior), the Hart line which is the area between the keratinized and unkeratinized labia epithelium (lateral), the fourchette (posterior), and the hymen (caudal). This structure is important for sexual stimulation and lubrication. Vulvar vestibulitis refers to increased sensitivity to pain at the opening of the vagina, making even gentle touch or stimulation painful [1]. Localized provoked vulvodynia (LPV) is the final term used for vestibulitis following consensus meetings [2]. Although the real incidence of LPV is not known due to the limited number of reports, its prevalence in the general population has been estimated as 10 to 28% [3]. It can affect women of all ages but usually develops between the ages of 20 and 50 years [4]. The exact etiology has not yet been fully elucidated, but to date, many pathophysiological mechanisms have been implicated. Specific disorders and factors associated with LPV include chronic inflammation, peripheral neuropathy, genetic, immunologic and hormonal factors, infectious processes, psychological disorders, sexual dysfunction, and central nervous system disorders. LPV is multifactorial, and different mechanisms have been proposed to clarify the etiopathogenesis of this disease. Some women with LPV may have a genetic predisposition associated with genetic polymorphism, prolonged inflammatory responses, and increased susceptibility to hormonal changes associated with oral contraceptive pills [5]. Increased inflammatory cells, mast cells, and subepithelial heparanase activity have been shown in LPV patients compared with controls [6,7]. Some researchers revealed altered microbiota, containing a lower level of lactobacilli and higher level of total fungi concentration [8] and suggested that abnormal vaginal microbiota was associated with the severity of LPV [9]. Since the 1980s, various dubious reports have also been published concerning the relationship between HPV and LPV. HPV was one of the implicated infectious agents but the presence of such a causal relationship could not be confirmed. Most authors, such as Wilkinson *et al*. (1993) suggested that LPV might be associated with an HPV infection in rare cases [10].

The treatment of LPV is extremely challenging since these patients seldom feel that they are completely cured. Comorbidities, such as fibromyalgia, interstitial cystitis, and back pain should be considered when planning the treatment. Furthermore, informing the patients about vulvar hygiene and care should be the first step, and a low oxalate diet with the daily supplementation calcium citrate could be beneficial. Medical therapy choices, including topical solutions (topical form of tricyclic antidepressants, lidocaine 5% and estrogen, compounded medications, such as topical amitriptyline 2% with baclofen 2%, capsaicin, cromolyn 4%, and nifedipine), oral agents (e.g. tricyclic antidepressants, selective serotonin reuptake inhibitors, venlafaxine, gabapentin, pregabalin, and carbamazepine), and injectable agents (e.g. steroids, bupivacaine, and interferon alpha) have been previously investigated [11]. Vaginal and external soft tissue mobilization and myofascial release, and biofeedback with surface electromyography and transcutaneous electrical nerve stimulation are among the physical therapy modalities for LPV, especially preferred in cases of pelvic floor muscle dysfunction. Muscle relaxants, such as valium suppositories, botulinum toxin, and biofeedback training are described as self-regulation strategies for confronting and reducing pain. Psychological interventions; e.g. sexual counseling and cognitive behavioral therapy reduce anxiety and fear related to dyspareunia in this group of patients. The surgical approach is offered to patients in case of the failure of the above-mentioned therapeutical methods for the management of LPV. Fenton's vestibulectomy is the most commonly performed surgery for LPV, which involves the excision of the posterior aspect of the vaginal vestibule. Moreover, the fascia posterior to the vagina is dissected until the fibers of the external anal sphincter are reached. Related studies reported the presence of HPV in the excised tissues, but none commented on the associated vulvar intraepithelial lesions. In this study, we aimed to investigate the histopathological results of 38 women with LPV, who underwent surgical vestibulectomy.

## Methods

In this retrospective study, fifty-five women (aged 19-45 years) that were admitted to a private gynecology clinic with LPV and underwent vestibulectomy between January 2016 and December 2018 were included. Universal Principles of the Helsinki Declaration were applied. Informed consent was obtained from patients/participants after explaining that participation in the study would not change the usual care according to the protocol. The LPV diagnosis was made using the modified Friedrich criteria: A history of vestibular pain upon touch or attempted penetration, tenderness to pressure localized within the vulvar vestibule on examination, and the exclusion of identifiable causes for the pain [12]. The diagnosis was confirmed through a Q-tip test, which was performed via pressing on the vestibular region with a cotton swab. The patients had a history of various medical therapies, but conservative treatments had failed to control their symptoms. The surgical intervention was performed under sedation anesthesia by a single surgeon. During vestibulectomy, the vulvar vestibule mucosa was excised between 1 and 11 o'clock, sparing the urethral orifice. The patients were invited for cognitive behavioral therapy at the fifth week, considering that physical problems could lead to psychological morbidities and hypertonus in pelvic muscles. After providing brief information about genital anatomy, physiology, and sexuality, the patients were engaged in dilator exercises, which were gradually increased in intensity. The histopathological results of 38 women were accessible and evaluated.

**Statistical analysis:** data analyses were performed by using Microsoft Excel 2010 for Windows. Results were expressed as percentages and mean ± SD.

## Results

**Figure 1:**
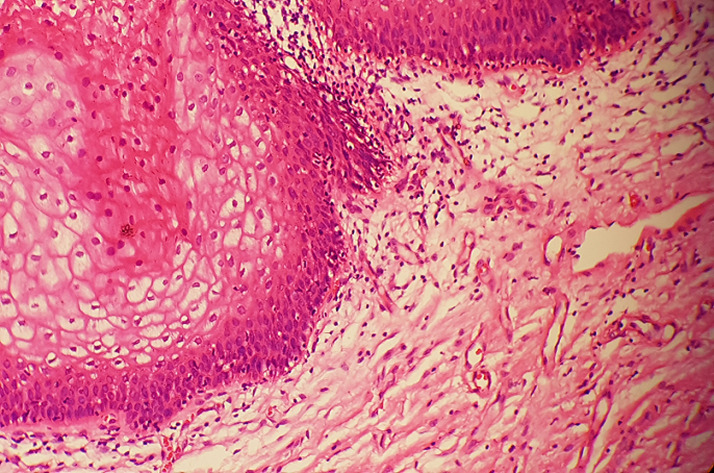
vulvar vestibulitis; edematous connective tissues, ectactic capillaries and mononuclear cells under vacuolized vestibular epithelium (H.E, x40)

**Figure 2:**
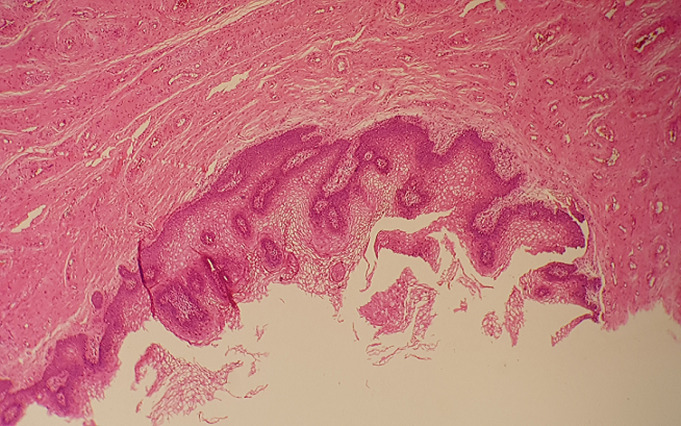
vulvar vestibulitis; diffuse inflammation findings in papillomatous connective tissue areas under thickened acanthotic epithelium (H.E, x10)

**Figure 3:**
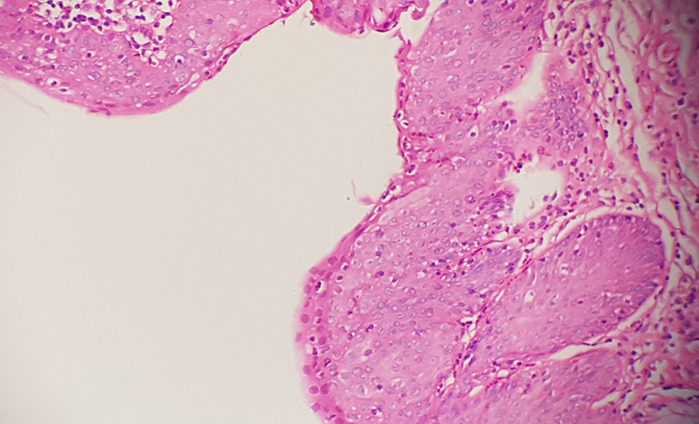
vestibular epithelium with widespread low-grade squamous intraepithelial lesion depending on HPV effect (H.E, x60)

**Figure 4:**
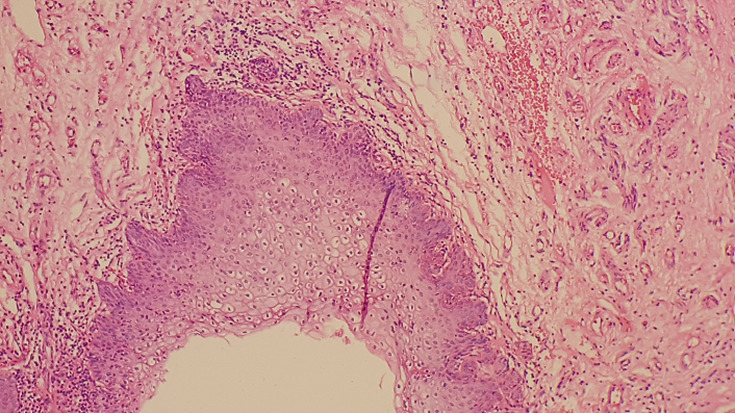
focal koilocytic atypia in squamous epithelium (H.E, x40)

The mean age of the patients was 31.4 ± 6.22 years. Fourteen (25%) of women were parous. Fourty (72%) of them were employed, 15 (27%) of them were housewives, 52 (94%) of them were married. Of the 55 women that underwent vestibulectomy, the histopathological results of 38 patients (69%) were available. In 21 of the 38 patients (21/38, 55.2%), the pathological reports revealed vestibulitis (Figure 1, Figure 2), whereas the findings were concordant with Low-Grade Squamous Intraepithelial Lesions (LGSIL) (Figure 3, Figure 4) for 14 cases (14/38, 36.8%). In three patients (7.8%), the pathology was reported as Lichen Simplex Chronicus (LSC). All the patients with an LGSIL were referred to a colposcopic examination and cervical cytology. The patients were asked to provide feedback via phone calls at three months of surgery. Fifty (90%) reported full recovery from dyspareunia after surgery and cognitive behavioral therapy. Five patients continued to have minimal pain but this did not affect their sexuality.

## Discussion

Although the treatment of LPV is usually based on clinical practice [13], all treatment options should be well known. A team approach is always encouraged with the possible combination therapies [14]. The British Society for the Study of Vulvar Diseases Guideline Group suggested that the surgical excision of the vestibule should be considered for only a minority of patients that have not responded to medical therapy [grade of recommendation B; evidence level IIb] [14]. However, the literature reports high satisfaction rates of vestibulectomy, ranging from 78 to 91% in women refractory to conservative management [15]. Therefore, surgical excision should be considered more frequently, rather than as a last resort. In the current study, the response to surgery was good in most patients, which is consistent with the literature [15-17]. Therefore, we consider that physicians, especially those that treat patients with LPV, should gain more experience about the surgical techniques involved. Another advantage of surgery is the opportunity to obtain histopathological results. The histological examination of the excised tissues provides an insight into the etiopathogenesis of the disease. In this study, the presence of an LGSIL in 36.8% of the excised tissues is an undeniable finding. The terminology regarding vulvar intraepithelial lesions has been extensively revised over years, but the most recent consensus in 2015 clarified the terms related to vulvar lesions. Since the Lower Anogenital Squamous Terminology (LAST) in 2012 only included HPV-associated lesions, ISSVD developed a new classification [18], in which vulvar lesions were grouped into LGSIL, high grade squamous intraepithelial lesion, and vulvar intraepithelial neoplasia. In previous definitions, LGSIL represented the HPV effect or flat condyloma, which caused many debates about prognosis. Although we know that these low-grade squamous lesions have less potential to progress into carcinomas, there is still an issue concerning their clinical significance.

Previously, Smith *et al*. reported a high frequency of vulvar dysplasia (10% vs. 0% in controls) in patients with LPV [19]. The authors calculated an age-adjusted odds ratio of 15.7 and discussed the possibility of these patients developing cancer unless they were treated for LPV. This was the first report that referred to the presence of LGSILs in the surgical specimens of the vestibule. Since LGSIL lesions are HPV-related, the association of LPV with an HPV infection should be the first possibility to be considered. However, the data about the role of HPV in LPV is inconsistent, and there are no up-to-date studies. In previous reports, the detection of HPV ranged from 5 to 100% [19], which raises questions concerning the reliability of the results. Srodon *et al*. detected a low-risk HPV infection in 67% of VIN1 cases and a high-risk HPV infection in 42% [20]. The authors concluded that low-grade lesions might contain high-risk HPV types. Another recent study investigated p16 expression in LGSIL but only 4% of the cases revealed staining with p16 [21]. Even so, the biological behavior of LGSIL cannot be predicted, and surgical excision probably contributes to the prevention of progression into invasive lesions. Another important finding of this study was the presence of LSC in three patients. LSC usually presents with itching and soreness and is mostly treated with topical steroids. The coexistence of LSC with LPV should never be underestimated as vulvar pain disorders are seen frequently. Therefore, it is recommended to exclude dermatological diseases before performing any surgical intervention. With a heterogeneous group of suggested etiologies, LPV might coexist with an HPV infection and/or LGSIL. Although this study is significant in terms of providing a new perspective based on the finding of 36.8% prevalence of LGSIL in patients with LPV, it also has some limitations such as small number of cases and absence of follow-up data.

## Conclusion

HPV infection or type in this group of patients would further support the findings of the study. Although the cause and effect relationship between HPV infections and LPV has not yet been proven, the current study indicates a possible correlation, which needs to be further elucidated with larger population studies. Furthermore, the current study suggests that before performing any surgical intervention as the final treatment, the patients diagnosed with LPV should be screened for HPV infections and preinvasive vulvar pathologies.

### What is known about this topic

- Vulvodynia is a chronic vulvar pain condition. Localised provoked vestibulo dynia (LPV) is the most common subset of vulvodynia, the hallmark symptom being pain on vaginal penetration;
- There are no specific tests. Diagnosis is made on the basis of a typical history, supported by examination findings and exclusion of other painful vulvar conditions;
- Surgical excision of the vestibule is usually considered for only a minority of patients with LPV that have not responded to medical therapy and association of HPV infection with LPV is inconsistent.

### What this study adds

- There is a 36.8% prevalence of low-grade squamous intraepithelial lesions in surgical vestibulectomy specimens of patients with localised provoked vestibulodynia;
- Screening for HPV infection and surgical vestibulectomy should be considered not only as a last resort.
